# Innovative Silver-Based Capping System for Mesoporous Silica Nanocarriers Able to Exploit a Twofold Anticorrosive Mechanism in Composite Polymer Coatings: Tailoring Benzotriazole Release and Capturing Chloride Ions

**DOI:** 10.1021/acsami.1c15231

**Published:** 2021-10-05

**Authors:** Federico Olivieri, Rachele Castaldo, Mariacristina Cocca, Gennaro Gentile, Marino Lavorgna

**Affiliations:** † Institute for Polymers, Composites and Biomaterials, National Research Council of Italy, Via Campi Flegrei, 34, 80078 Pozzuoli, Italy; ‡ Institute for Polymers, Composites and Biomaterials, National Research Council of Italy, P.le E. Fermi 1, 80055 Portici, Italy

**Keywords:** mesoporous materials, nanoparticles, coatings, corrosion, smart release

## Abstract

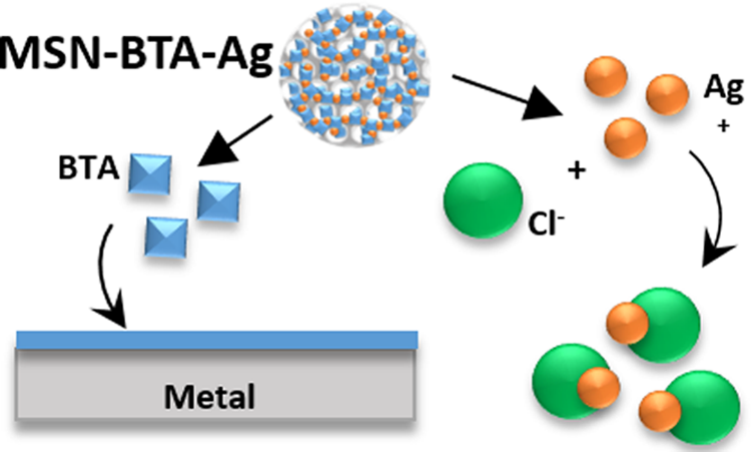

In this work, engineered stimuli-responsive mesoporous silica nanoparticles (MSNs) were developed and exploited in polymer coatings as multifunctional carriers of a typical corrosion inhibitor, benzotriazole (BTA). In detail, a new capping system based on a BTA–silver coordination complex, able to dissolve in acid and alkaline conditions and to simultaneously tailor the BTA release and the capture of chloride ions, was properly designed and realized. Acrylic coatings embedding the engineered MSNs were deposited onto iron rebar samples and tested for their protective capability in acid and alkaline environments. Results highlighted the high potential of the proposed system for the protection of metals, due to the synergistic effect of the mesoporous structure and the capping system, which guaranteed both the sequestration of chloride ions and the on-demand release of the effective amount of anticorrosive agents able to ensure the enhanced protection of the substrate.

## Introduction

Protection from corrosion mechanisms is one of the most relevant objectives for the improvement of the durability of civil and industrial structures made of both metals and metal-reinforced concretes. Several strategies are employed to prevent or slow down the corrosion of these structures, starting from the optimized design and choice of protective materials to including periodic maintenance interventions. In this context, the application of polymer-based protective coatings onto both metals and concrete surfaces is a strategy widely exploited to prolong the service life of existing structures.^1−3^ The effectiveness of a protective coating can be highly enhanced by its doping with smart systems based on corrosion inhibitors, which protect metal and concrete structures by tailoring the release of anticorrosive additives under specific degradation conditions^4−7^ and by preventing the UV-induced deactivation of anticorrosion agents.^6^

In particular, nanocomposite coatings embedding nanocarriers of active molecules represent a highly appealing approach.^8−12^ Promising nanocarrier candidates are, for instance, micro or nanocapsules,^13,14^ inorganic nanotubes (i.e., halloysite),^15^ and inorganic nanoparticles.^16−19^ Among the latter, mesoporous silica nanoparticles (MSNs) are very attractive due to their easy preparation, high chemical stability and resistance, and high porosity and surface area.^1,20−22^ Indeed, MSNs have been already exploited as nanocarriers of corrosion inhibitors^9,14,23−25^ and, even more, as smart stimuli-responsive nanocarriers,^26−29^ able to release active molecules in response to external triggers.^30−32^

Various approaches are aimed at developing smart stopping systems: a widely employed strategy is based on the formation of a coordination complex between 1,2,3-benzotriazole (BTA), a typical anticorrosion agent, and copper ions on the external side of high-surface-area nanocarriers. Lvov et al.^15,33,34^ first exploited this mechanism by loading halloysite nanotubes with BTA and inducing the formation of BTA–copper complexes at the edges of the inorganic nanotubes. In a recent work, Castaldo et al.^9^ exploited the same stopping system applied on MSNs. The BTA–copper coordination complex was found to be an effective stopping/tailoring release system also for MSN, allowing the release of the anticorrosive agent over a wide acid exposure range.

Based on this background, we aimed at developing an alternative BTA stopping/tailoring release system based on a different coordination complex. Our attention was focused on silver, another metal able to form chelating complexes with BTA,^35,36^ in which silver ions form a coordination net that may act as a stopper, tailoring the release of active compounds from porous structures.

An advantage of silver with respect to copper is the limited solubility of some silver salts, which allows to design a long-lasting stimuli-responsive release system. Moreover, the BTA–silver complex attains a twofold function system: when the BTA–silver complex is slowly dissolved and the corrosion inhibitor is released, free silver ions are available to exploit a capture mechanism toward specific anions, such as chloride ions, responsible for typical corrosion mechanisms in metal structures. In fact, chloride ions can penetrate the passive layer, accelerating the oxidation of metal alloys and forming corrosion pits on metallic substrates, owing to their high electrochemical potential and reactivity.^37^

Moreover, despite its efficiency, the MSN–BTA system capped with copper ions showed an aesthetic drawback: the BTA–copper complex is characterized by a typical green–blue color, which limits its use for applications in which the protective coatings should not induce color changes, such as cultural heritage applications.^38^ On the contrary, the BTA–Ag coordination complex is white, a more neutral color for the above-mentioned applications.

Thus, the effectiveness of the BTA–silver complex was assessed using MSNs as nanocarriers, which are also able to protect BTA from the degradation caused by UV irradiation.^9^ MSNs loaded with BTA and capped with silver ions showed a pH-dependent release of the anticorrosive agent, negligible at neutral pH and faster at low and high pH values, where the complex is slightly soluble. The capability of the co-released silver ions to capture chloride ions when the complex dissolves was also demonstrated. Finally, the innovative corrosion inhibitor nanocarrier system was validated in an acrylic polymer coating, applied for the protection of iron rebars. Loading the anticorrosive agent into the MSNs allowed embedding in the coatings a certain amount of active agents, which were slowly released when necessary, i.e., under an applied stimulus, and therefore in a gradual way. This avoided the formation of BTA aggregates, which would eventually limit the anticorrosion efficacy of active agents.

## Experimental Section

### Materials

Tetraethyl orthosilicate (TEOS), cetyltrimethylammonium bromide (CTAB), triethanolamine (TEAH_3_), 1,2,3-benzotriazole (BTA), silver(I) sulfate (Ag_2_SO_4_), hydrochloric acid (HCl, assay 36%), and ethanol were purchased from Sigma–Aldrich (Milan, Italy). Bidistilled water was also used for all of the laboratory procedures. Iron-based disks were used as mock-up metal substrates (diameter ≈ 1.2 cm, thickness ≈ 0.7 cm). An acrylic resin dissolved in xylene (dry content 20–25 wt %) containing 1.5 wt % of benzotriazole (BTA) with respect to dry content, commercial name Metacril, was obtained from Antichità Belsito (Rome, Italy).

### Preparation of the BTA–Ag Complex

Overall, 2.0 g of BTA was dissolved in 100 mL of distilled water and was treated with an excess of Ag_2_SO_4_ (about 4.0 g). The precipitated white BTA–Ag complex (C_6_H_4_N_3_^–^Ag^+^ in eq 1) was collected through filtration, repeatedly washed with water (about 2 L) to dissolve the eventual excess of BTA, and dried at room temperature overnight. About 3.76 g of BTA–Ag was obtained.
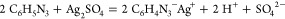
1

### Synthesis of MSN

CTAB, TEAH_3_, and bidistilled water were stirred for 1 h at 80 °C. Then, TEOS was quickly added in the mixture, which was stirred for further 2 h. CTAB, TEAH_3_, H_2_O, and TEOS were included in a 0.06:8:80:1 relative molar ratio. After the hydrolysis and condensation of TEOS, MSNs were formed and collected through filtration, washed with water, and calcined at 600 °C for 6 h.

The yield of the reaction was evaluated as follows: the molecular weight of MSN was approximated to the molecular weight of SiO_2_ (60.08 g/mol), omitting the contribution of terminal hydroxyl groups, and based on stoichiometry, the yield of reaction (*Y*) was found through eq 2
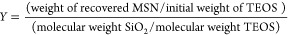
2

### BTA Loading into MSNs

A solution containing 4.8 g of BTA dissolved in 60 mL of acetone at room temperature was adsorbed by MSNs, previously dried under vacuum overnight. The loading procedure was divided into 6 loadings of 10 mL of solution to increase the loading efficiency. MSNs loaded with BTA were then treated with distilled water at 40 °C for 1 h under stirring to remove the excess of BTA deposited onto the surface of porous inorganic particles. These nanoparticles were centrifuged at 13 500 rpm for 10 min at 40 °C, frozen using liquid nitrogen, and freeze-dried overnight. These nanoparticles were called MSN–BTA.

### Silver-Based Capping Layer onto MSNs Filled with BTA

One gram of MSN–BTA was immersed into 200 mL of a water solution of silver sulfate (20 mM), kept under mechanical stirring for 2 min, and then centrifuged at 13 500 rpm for 10 min. The obtained nanoparticles, coded MSN–BTA–Ag, were collected through filtration, washed with water, separated by centrifugation, frozen in liquid nitrogen, and freeze-dried.

### Preparation of Anticorrosion Coatings

Iron disks as metal mock-up samples were lapped with SiC grinding paper, grit size P600, to obtain a smooth surface.

MSN–BTA–Ag were ultrasonicated and dispersed in a 1.5 wt % Metacril in xylene solution at 3 wt % with respect to the dry content of the solution with a Sonics Vibracell ultrasonic processor (Newton), at 25% of amplitude (500 W, 20 kHz) for 10 min, with 15/15 s on/off cycles, while the dispersion was kept in an ice-water bath, to avoid its overheating. This mixture was deposited by drop-casting onto a series of iron disks to obtain coatings of about 2 μm thick. The iron disks coated in this way were coded ACR–MSN–BTA–Ag and the applied coating contained an overall amount of 2 wt % of BTA with respect to the dry content of the acrylic resin (1.5 wt % in the commercial formulation + 0.5 wt % corresponding to the BTA from the MSN–BTA–Ag).

To compare the anticorrosive effect of the smart nanocarriers with respect to free BTA, iron disks were also coated with a Metacril solution containing additional BTA in the same amount as the BTA included in the MSN–BTA–Ag embedded in the ACR–MSN–BTA–Ag coating (0.5 wt % of additional BTA with respect to the dry content of the product). These disks were coded ACR–BTA and contained an overall amount of 2 wt % of BTA with respect to the dry content of acrylic resin (1.5 wt % in the commercial formulation + 0.5 wt % of free additional BTA).

Further iron disks were coated with Metacril containing the BTA–Ag complex and an overall amount of 2 wt % of BTA, coded ACR–BTA–Ag. In this case, the total amount of BTA consisted of 1.5 wt % free BTA from the commercial formulation and 0.5 wt % of BTA from the BTA–Ag complex.

Two additional disks were coated with an overall amount of free BTA of 3 and 4 wt % with respect to the dry content of the acrylic resin, coded respectively ACR–BTA-3 and ACR–BTA-4, to verify the effect of the BTA amount on anticorrosive efficiency of coatings. For comparison, iron disks were also coated with pristine Metacril, and these disks were coded ACR.

Before testing, all of the iron disks were dried at room temperature for at least 48 h, to ensure complete solvent evaporation.

ACR–BTA, ACR–BTA-3, ACR–BTA-4, and ACR–MSN–BTA–Ag coatings were also prepared onto glass substrates to study how the coating morphology is affected by the BTA concentration and exclude possible defects due to iron substrate roughness.

### Characterization

Bright-field transmission electron microscopy (TEM) analysis was performed on MSN, MSN–BTA, and MSN–BTA–Ag. The nanoparticles were dispersed by mild sonication in ethanol with a Sonics Vibracell (Newtown, CT) ultrasonic processor (500 W, 20 kHz) at 25% of amplitude for 5 min, and then carbon-coated copper grids were immersed into the dispersions and dried at room temperature. TEM analysis was performed using a FEI Tecnai G12 Spirit Twin (LaB_6_ source) at 120 kV acceleration voltage (FEI, Eindhoven, The Netherlands), and TEM images were taken with a FEI Eagle 4 k CCD camera.

Nitrogen adsorption analysis was performed on MSN and MSN–BTA–Ag using a Micromeritics ASAP 2020 analyzer (Norcross, GA) and Micromeritics MicroActive software for data evaluation. N_2_ adsorption/desorption isotherms were registered at 77 K, and specific surface area (SSA) was determined using the Brunauer–Emmett–Teller (BET) equation over the standard BET range (*p*/*p*_0_ = 0.05–0.3). Total pore volume and pore size distribution were evaluated through the nonlocal density functional theory (NLDFT) using a model for oxide surfaces with cylindrical pores with a 0.1 regularization over the adsorption branch of the isotherm. The adsorption measurements were performed using high-purity gases (>99.999%); samples were degassed at 100 °C under vacuum before analysis (*P* < 10^–5^ mbar).

Thermogravimetric analysis (TGA) for MSN, MSN–BTA, and MSN–BTA–Ag particles was performed by heating the samples from 25 to 800 °C at a rate of 20 °C/min in oxidative conditions, using a PerkinElmer Pyris Diamond TG/DTA (Waltham, MA). Energy-dispersive X-ray (EDX) analysis was also performed on MSN–BTA–Ag and pristine MSN, to confirm the loading of the nanoparticles, by means of a FEI Quanta 200 FEG scanning electron microscope (SEM, FEI, Eindhoven, The Netherlands) equipped with an Inca Energy System 250 and an Inca-X-act LN2-free analytical silicon drift detector (Oxford Instruments, Abingdon-on-Thames, UK).

The BTA–Ag complex obtained by precipitation of BTA with Ag^+^ was analyzed by scanning electron microscopy by means of the above-mentioned SEM in high vacuum mode. The precipitated was deposited in a powder form onto an aluminum stub, sputter coated with a thin layer of Au/Pd, and observed with a secondary electron detector. Moreover, the BTA–Ag complex was analyzed by Fourier transform infrared (FTIR) spectroscopy in attenuated total reflectance (ATR) mode, using a PerkinElmer Spectrum One (Waltham, MA) FTIR spectrometer equipped with an ATR module, using a resolution of 4 cm^–1^ and 32 scan collections.

The solubility of the BTA–Ag complex at different pH values was evaluated by dissolving the BTA–Ag-based complex at 25 °C in buffer solutions and evaluating the amount of dissolved BTA by UV–vis spectroscopy analysis using a Jasco V570 UV spectrophotometer (Jasco, Easton, MD). In detail, 2.2 mg of BTA–Ag was dissolved in 10 mL of buffer solutions at pH 5, 6, 7, 8, and 9. After 24 h, the dissolution was evaluated by UV–Vis spectroscopy using calibration curves collected at acid, neutral, and basic pH values on pristine BTA solutions.

The kinetics of BTA release from the smart nanocarriers MSN–BTA–Ag was evaluated in water at pH 7.0 and in HCl (pH 1.5, 4, and 5.5) and NaOH (pH 8.5, 10.0, and 12.5) aqueous solutions. Tests were performed at 25 °C. MSN–BTA–Ag were dispersed in the test solutions at a concentration of 0.015 mg/mL, and the release of BTA was monitored by measuring at constant time intervals the BTA concentration of the solution through UV spectroscopy. BTA calibration curves in water, HCl, and NaOH solutions were reliably collected. After the release tests performed with HCl at pH 1.5, SEM and EDX analyses were performed on the precipitated salt by means of the above-mentioned SEM-EDX equipment. Also, wide-angle X-ray diffraction (XRD) analysis was performed on the precipitate by means of a Panalytical X’Pert Pro diffractometer equipped with a PixCel 1D detector using Ni-filtered Cu Kα_1_/Kα_2_ radiation generated at 40 kV and 40 mA. The precipitate was collected by filtration and dried under vacuum before analysis.

EDX analysis was performed on the iron disks to characterize their elemental composition. Then, iron disks coated with ACR–MSN–BTA–Ag, ACR–BTA, ACR–BTA–Ag, ACR–BTA-3, ACR–BTA-4, and ACR coatings were subjected to accelerated corrosion tests by (i) exposure to vapors of a 1.5 pH HCl solution at 60 °C for a total of 18 h and (ii) direct contact with a 12.5 pH NaOH solution for a total of 3 h at 40 °C. The evolution of corrosive phenomena onto the disks was monitored by a Lynx EVO stereomicroscope (Vision Engineering Ltd, Milan, Italy). In the case of direct contact with NaOH solution, before microscopy observation of the disks’ surface, the samples were washed with distilled water and dried with filter paper. The quantification of the corroded areas was evaluated through ImageJ software with a consolidate procedure.^6^ After the corrosion tests, SEM analysis was performed onto the corroded disks through the above-mentioned equipment. Untreated ACR–BTA, ACR–BTA-3, and ACR–BTA-4 were also analyzed by SEM for comparison.

## Results and Discussion

First, the novel BTA–Ag complex was prepared by dissolution of BTA in water and the subsequent addition of a silver ions water solution. The stoichiometric analysis of the collected precipitate indicated an approximate BTA/Ag ratio of 1:1. SEM analysis of the BTA–Ag complex (Figure S1) revealed that, in stark contrast to the morphology of pristine BTA, constituted by very large (millimeter sized) needle-shape crystals, the precipitated complex is constituted by platelets with an irregular shape, with a lateral size ranging in a quite narrow range, approximately between 0.3 and 2.0 μm. FTIR analysis of the complex confirmed the occurrence of the chemical bond between BTA and silver ions. The FTIR spectrum of the BTA–Ag complex (Figure S2) showed the absorption bands typical of the C=C stretching (weak) in ortho-disubstituted aromatic rings, centered at 1580–1470 cm^–1^, and the bands corresponding to out-of-plane C-H bending (strong) in the 780–700 cm^–1^ region. The absorption bands of the triazole ring (C=N and N=N, medium/weak) were found centered in the region of 1450–1120 cm^–1^. The strong absorption band centered at 1143 cm^–1^ was attributed to the formation of Ag(I)/N interactions in the BTA–Ag complex, as a consequence of the shift of the N–N–N stretching band originally centered at 1204 cm^–1^ in the pristine BTA.^39,40^

The results of solubility tests of the BTA–Ag complex at different pH values revealed the pH-dependent solubility of the complex, low in all of the investigated pH ranges (5–9), with a minimum at pH 7–8 and slightly higher at pH equal to or lower than 6 and equal to or higher than 9 (Figure S3).

**Figure 1:**
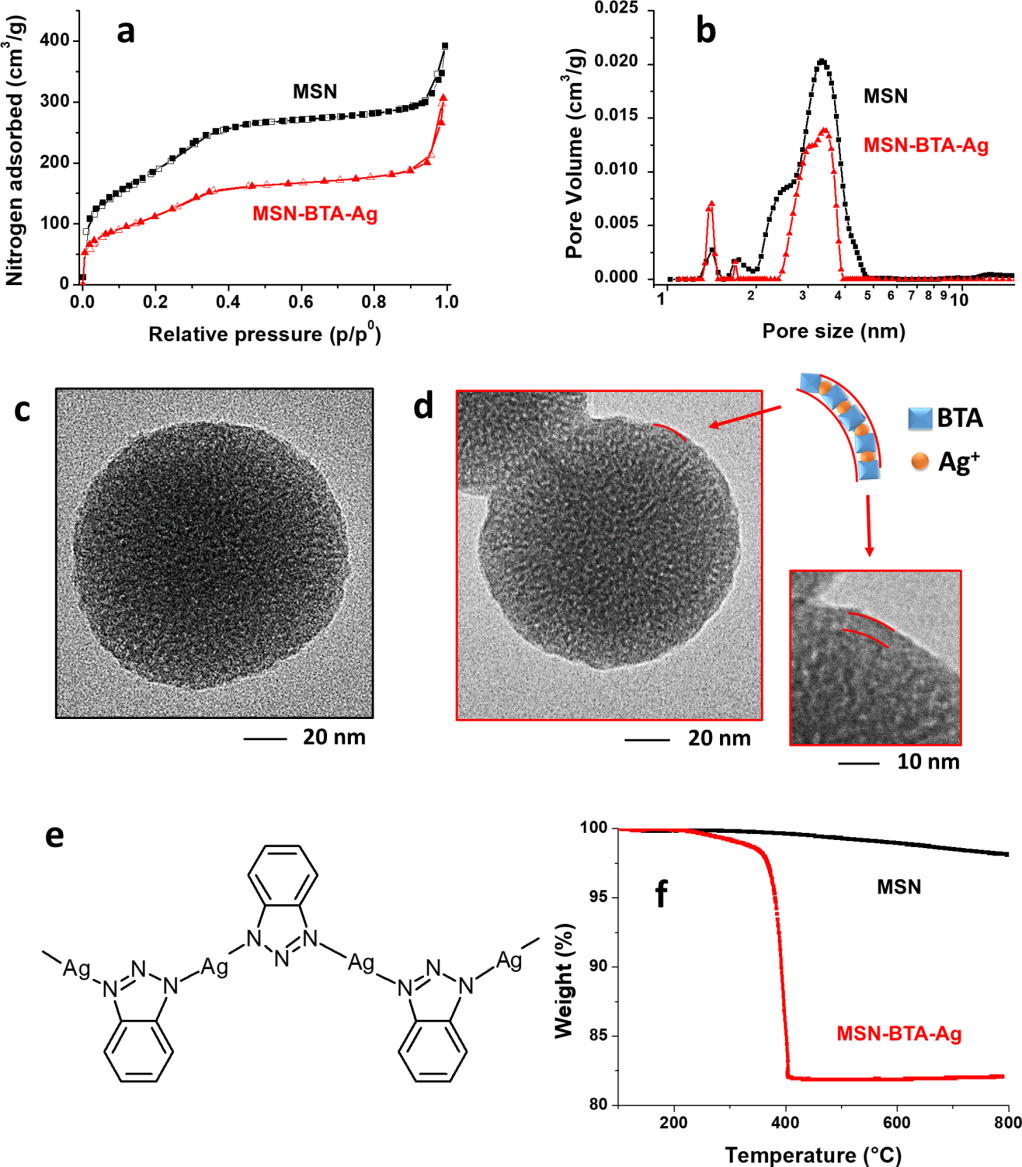
Nitrogen adsorption/desorption isotherms (a, adsorption in full symbols and desorption in hollow symbols) and NLDFT pore size distribution (b) of MSN (black squares) and MSN–BTA–Ag (red triangles); TEM images of MSN (c) and MSN–BTA–Ag (d) with schematic and chemical (e) representation of the BTA–Ag capping layer; TGA analysis of MSN (black squares) and MSN–BTA–Ag nanoparticles (red triangles) (f).

Then, BTA was loaded in MSN nanocarriers and the novel BTA–Ag capping system was applied, realizing the MSN–BTA–Ag system. With this aim, MSN were synthesized through a facile high-yield/high-throughput procedure.^41^ Indeed, the yield of the MSN synthesis was about 98%, as evaluated by eq 2 reported in the Experimental Section. The mesoporosity of the obtained nanoparticles was evaluated through nitrogen adsorption analysis. The N_2_ isotherm of the MSNs is a type IV isotherm (Figure 1a), characteristic of materials with ordered porosity, with pronounced adsorption at very low relative pressure due to the presence of a fraction of smaller mesopores. Indeed, NLDFT pore size distribution (Figure 1b) shows a major pore size distribution with the main peak at around 3.3 nm and a minor shoulder at 2.5 nm. MSNs are characterized by a BET SSA of 700 ± 14 m^2^/g and a total porosity of 0.47 cm^3^/g. MSN–BTA–Ag were also analyzed by nitrogen adsorption, showing a clear reduction of the accessible porosity and specific surface area with respect to the pristine MSN, which are reduced respectively to 0.31 cm^3^/g and 430 ± 9 m^2^/g. This porosity and specific surface area reductions found in MSN–BTA–Ag particles with respect to the pristine MSN are in accordance with the filling of the nanocarriers porosity with the corrosion inhibitor BTA and the BTA–Ag capping complex.

The ordered porous structure of MSNs is confirmed by TEM analysis, which reveals the MSNs’ typical worm-like structure (Figure 1c,d). From image analysis, the MSN diameter was evaluated to be 90 ± 25 nm (average value and standard deviation), with all particles’ size ranging from about 50 to 140 nm. The formation of the BTA–Ag complex does not significantly alter the morphology of MSNs. Indeed, only a thin dense layer can be observed on the MSN–BTA–Ag external surface (Figure 1d), which can be attributed to the BTA–Ag complexes accumulated in the outer shell of the nanocarrier. As previously reported, studies on BTA–metal complexes have led to the identification of an energetic favorable “polymeric” BTA–Ag structure with silver connected to two N atoms (Figure 1e).^42,43^

EDX analysis (Table S1) confirmed the presence of BTA (due to carbon) and silver in MSN–BTA–Ag. A quantitative evaluation of the amount of BTA loaded into the MSN–BTA–Ag particles was obtained by comparative analysis of MSN and MSN–BTA–Ag TGA traces (Figure 1f). Indeed, upon heating in oxidative conditions, MSN–BTA–Ag shows a significant degradation with an onset at about 200 °C up to about 400 °C, due to the thermo-oxidative degradation of BTA. MSN–BTA–Ag show, at the end of the analysis, a residual weight of about 82% of the pristine weight of the sample. MSNs, on the other hand, show only a reduced degradation starting at about 320 up to 800 °C, due to the hydrogen-bonded hydroxyls released and presents, therefore, only a slightly reduced weight at 800 °C of about 98% of the pristine MSN weight. Thus, by comparison of the TGA residual weight of MSN and MSN–BTA–Ag samples, the amount of BTA loaded into MSN–BTA–Ag is estimated to be about 16 wt %. Considering the BTA density (1.36 g/cm^3^), this amount corresponds to a filling with BTA of about 30% of the available MSN pore volume, in good agreement with nitrogen adsorption analysis results.

As hypothesized, the formation of the BTA–Ag complex does not alter the neutral coloration of MSNs, differently from the typical blue color of the BTA–Cu complex and the corresponding MSN–BTA–Cu system9 (Figure S4).

**Figure 2:**
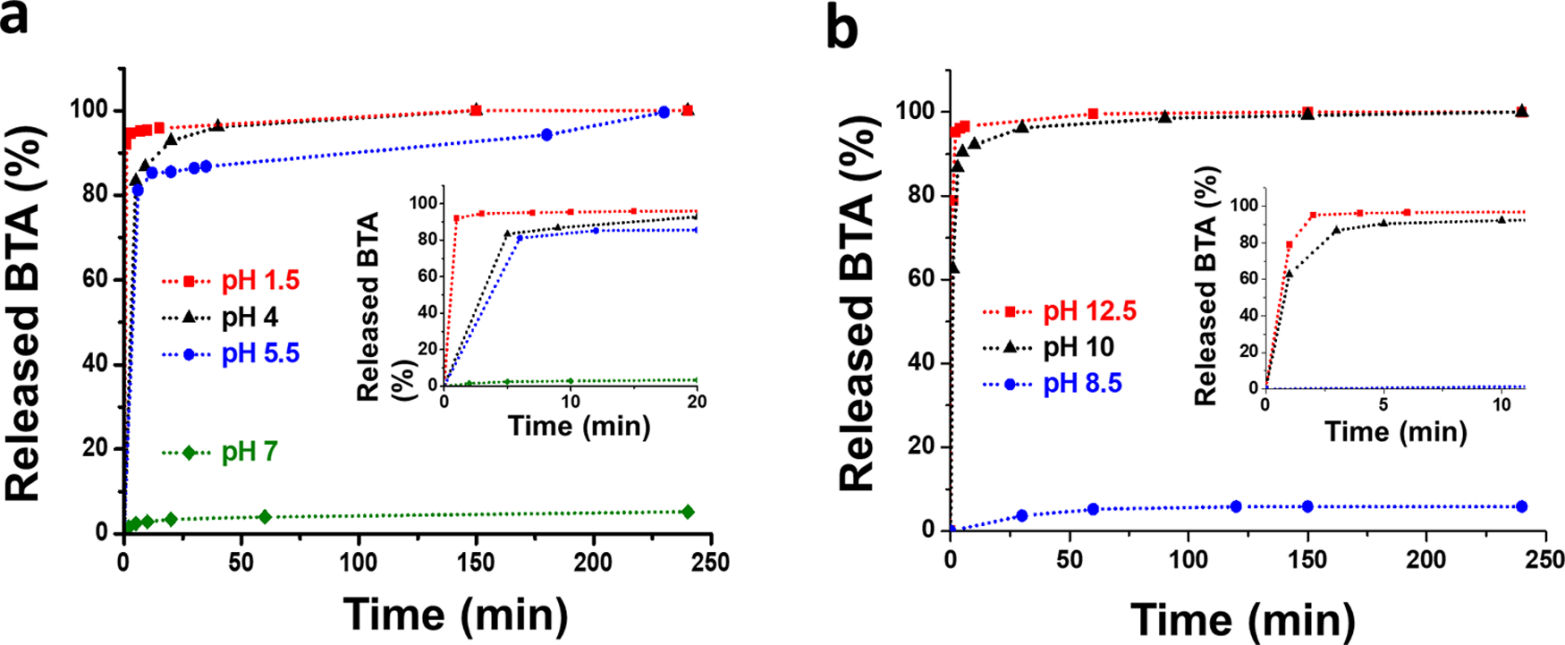
BTA release from MSN–BTA–Ag in (a) neutral and acid conditions and (b) alkaline conditions.

BTA release tests from MSN–BTA–Ag were performed in acid and basic conditions to characterize the kinetics of the BTA release from the nanocarriers at different pH values, which simulate different exposure conditions. Only 4 wt % of the loaded BTA is released in water at pH 7 (Figure 2a) also after prolonged release experiments. On the contrary, the BTA release is faster in all of the acid conditions tested. Indeed, 92 wt % of BTA is released in 1 minute at pH 1.5 and in 20 min at pH 4.0. In both cases, complete release of BTA is achieved within about 2 h. At pH 5.5, BTA release is slightly slower; about 86 wt % of BTA is released in 20 min and the complete release is achieved in about 4 h (Figure 2a). In basic conditions (Figure 2b), a very fast release of BTA is observed at pH 10.0 and 12.5, with 95 wt % of BTA being released in 2 min at pH 12.5 and 90 wt % of BTA being released in 5 min at pH 10.0. In both cases, BTA is completely released within 150 min. On the other hand, the BTA release is low in moderately basic conditions (pH 8.5), where only about 6 wt % of BTA is released after 150 min (Figure 2b).

This trend clearly indicates that the release mechanism of BTA is strictly correlated to the solubilization of the BTA–Ag complex. At neutral or slightly basic pH, the nearly insoluble BTA–Ag exploits its capping ability, physically occluding the particle surface and preventing the release of the free BTA loaded in the internal volume of the MSNs. On the contrary, at pH values far from neutral or slightly basic conditions, i.e., when the BTA–Ag complex mainly located on the surface of the MSNs starts to be dissolved, the free BTA loaded in the internal volume of the MSNs is progressively released.

**Figure 3:**
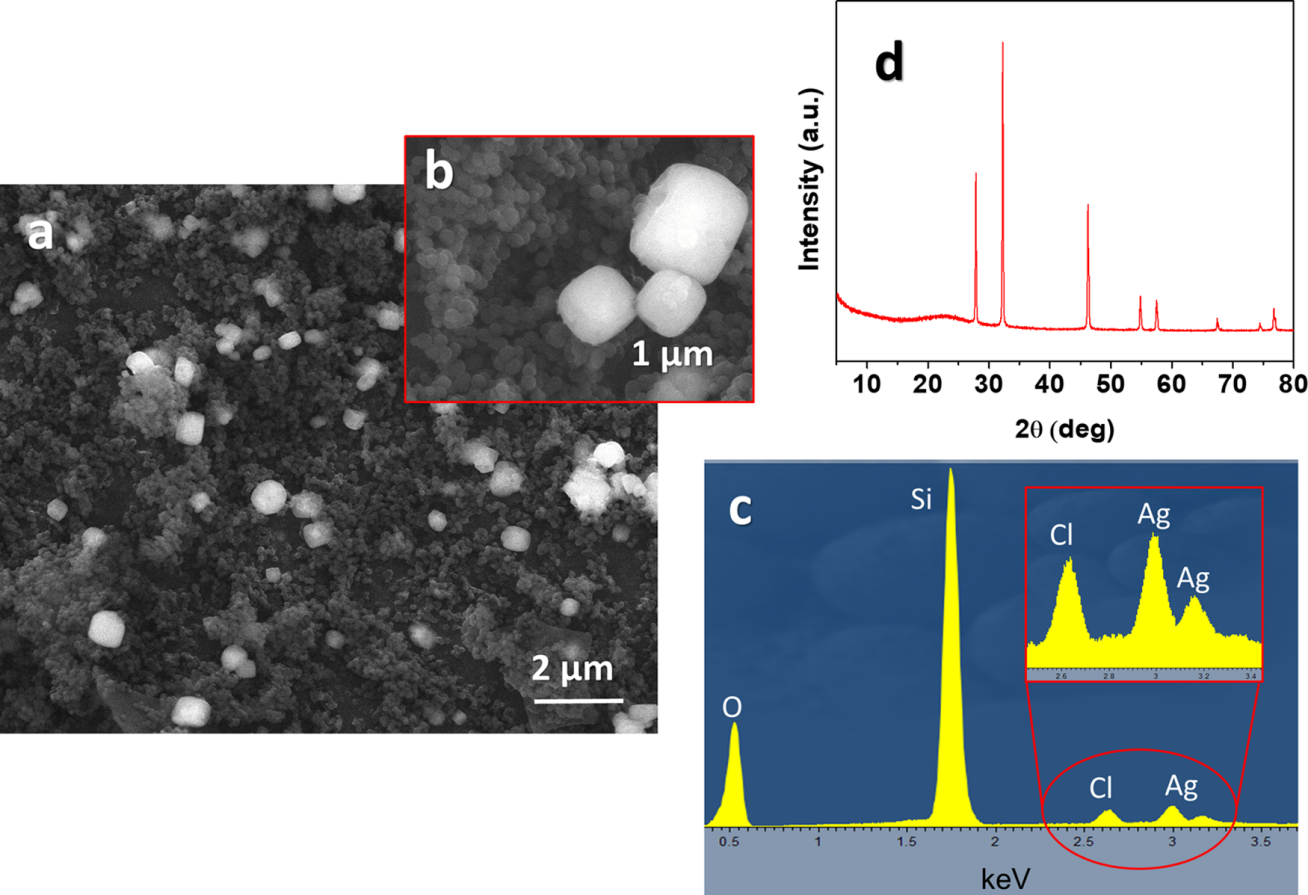
(a, b) SEM images and (c) EDX, and (d) XRD results of the precipitate collected after the release test of BTA from MSN–BTA–Ag performed in acid conditions with HCl (pH 1.5).

The nanoparticles and the precipitate collected after the release tests performed in acid conditions with HCl (pH 1.5) were analyzed by SEM, EDX, and XRD analyses. The results showed a large presence of cubic-shaped crystals among the MSNs (Figure 3a,b). EDX analysis (Figure 3c) revealed the presence of Si, O, Ag, and Cl in the sample, with Cl and Ag in the 0.96 ± 0.06 molar ratio, indicating that, upon release of BTA, the Ag^+^ ions previously coordinated to BTA are able to react with the Cl^–^ ions, with consequent AgCl precipitation. The XRD spectrum (Figure 3d) reveals the characteristic AgCl pattern composed of the diffraction peaks at 27.29, 32.23, 46.24, 52.84, 57.49, 67.46, 74.51, and 76.73° associated respectively with the (111), (200), (220), (311), (222), (400), (331), and (420) AgCl planes.^44^ Also, the diffraction peak at 22° is attributed to amorphous silica (MSNs).^45^ This phenomenon is attributed to the very low solubility of AgCl, which at 25 °C exhibits a solubility product of 1.8 × 10^–10^ mol^2^/L^2^;^46^ this value means that only about 1.9 mg of AgCl is dissolved per liter of water.

**Figure 4:**
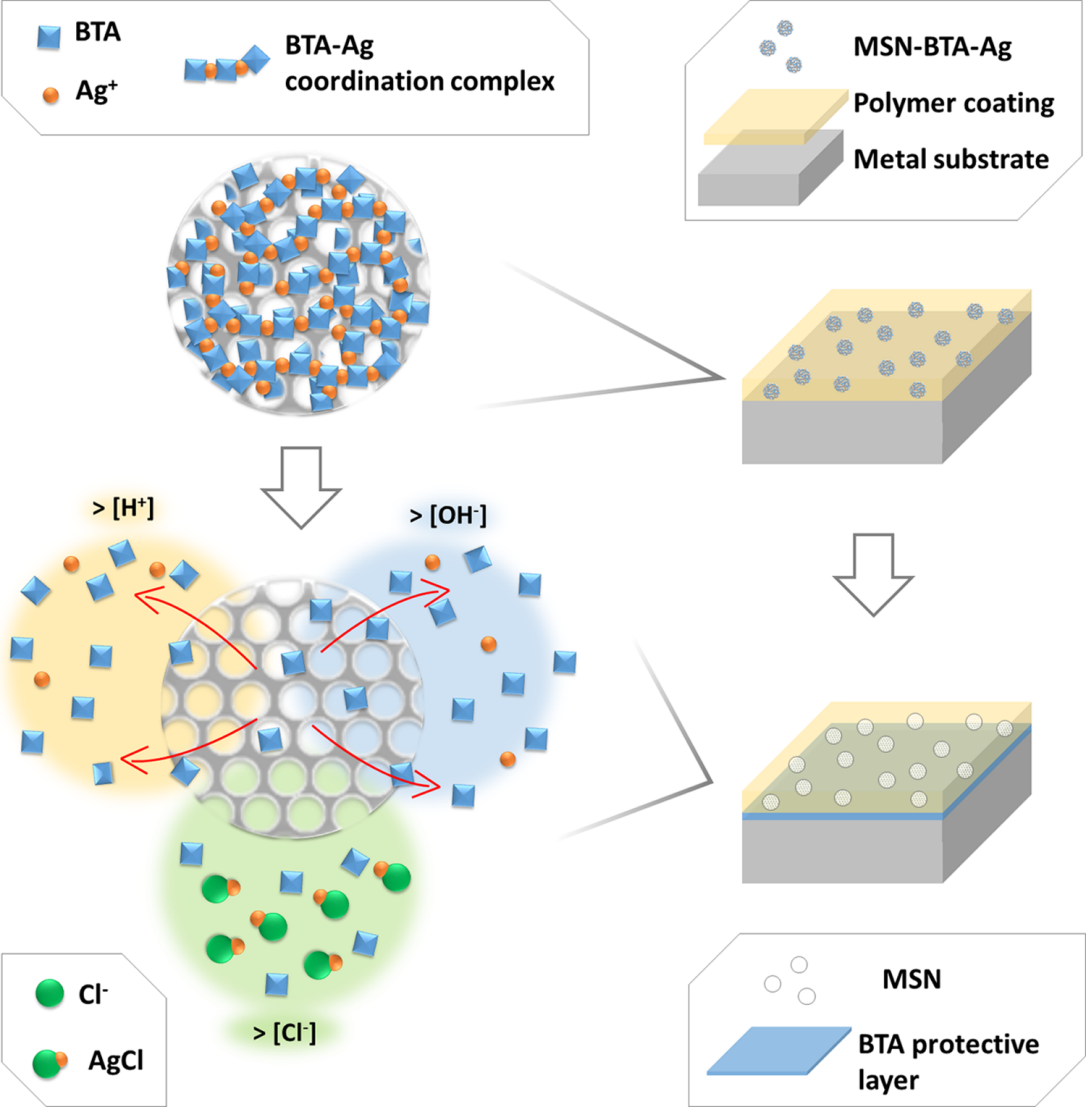
Representation of MSN–BTA–Ag anticorrosive mechanisms. In conditions far from neutrality, the BTA–Ag complex solubilizes and the BTA loaded in the internal volume of MSN is released from the silica mesopores, creating a passivating layer on the metal substrate. Moreover, in presence of chloride ions, Ag^+^ reacts with Cl^–^, precipitating as AgCl.

Therefore, the BTA–Ag coordination complex employed as a stopping system in the MSN–BTA–Ag smart nanocarriers forms a capping layer that acts as a pH-regulated net, tailoring the release of the active compounds depending on the pH of the environment. In particular, the release of BTA from MSN is inhibited in neutral conditions while it is modulated in response to acid or alkaline environmental stimuli. Moreover, linked to the BTA release is the capture of chloride ions, responsible for typical corrosion mechanisms in metal and metal-reinforced concrete structures.^38^ In this way, the MSN–BTA–Ag nanocarriers are able to ensure both passive and active protection from corrosion in aggressive conditions involving Cl^–^, by releasing BTA, which creates a protective layer adsorbed on the metal surface,^47−49^ and by releasing the silver ions, which neutralize the chloride ions through the formation of the AgCl precipitate. In Figure 4, the multiple anticorrosive mechanisms of MSN–BTA–Ag nanoparticles are schematized.

The corrosion inhibition efficiency of the MSN–BTA–Ag particles was evaluated by accelerated corrosion tests for iron rebar samples coated with a polymeric 2 μm thin film containing the smart nanocarriers. The elemental composition of the iron disks was measured by EDX analysis, which revealed the presence of Fe (93.4 wt %), C (4.3 wt %), Mn (1.1 wt %), Si (0.7 wt %), and Cu (0.5 wt %). An optical image of an untreated iron disk is reported in Figure S5.

The polymer coating chosen is a commercial product largely used in cultural heritage applications (Metacril), based on poly(ethylacrylate-*co*-methylmethacrylate) containing 1.5 wt % of free BTA with respect to the acrylic resin content. This approach was selected because it is generally accepted that to maximize the effect of a smart nanocarrier containing an anticorrosion agent, the smart system must be added to a product that contains a certain amount of free anticorrosion agents.^27,50−52^ The free fraction of the anticorrosion agents is able to improve the protection, whereas the nanocarrier acts as a reservoir of further anticorrosion agents, able to enhance and extend the protective effect of the coating and also protect the anticorrosion agents from UV degradation.^6^ As detailed in the Experimental Section, the amount of MSN–BTA–Ag particles added to the commercial product was 3.0 wt % with respect to the acrylic resin content, corresponding to an additional 0.5 wt % of BTA. Applying this formulation onto iron mock-up samples, the coated ACR–MSN–BTA–Ag samples were obtained. Disks coated with similar amounts of the neat commercial coating (coded as ACR) and the commercial product containing additional 0.5 wt % of corrosion inhibitor dissolved (coded as ACR–BTA), thus with a total amount of BTA equal to that contained in ACR–MSN–BTA–Ag samples, were tested by comparison.

**Figure 5:**
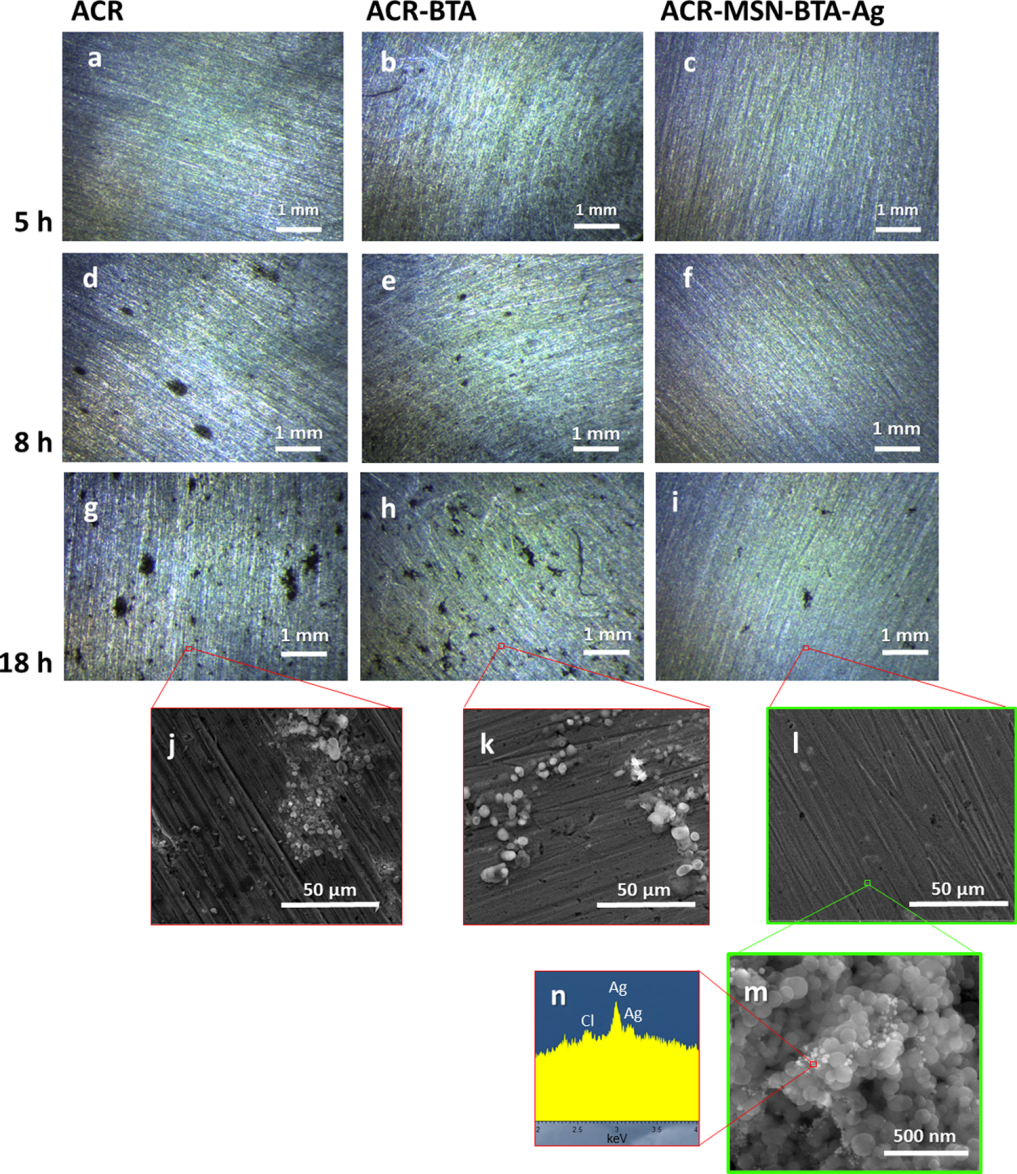
Optical images of iron rebar disks coated with (a, d, g) commercial acrylic resin ACR; (b, e, h) commercial resin loaded with additional free BTA (ACR–BTA); (c, f, i) commercial resin loaded with the smart MSN–BTA–Ag system (ACR–MSN–BTA–Ag) after (a–c) 5 h, (d–f) 8 h, and (g–i) 18 h of exposition to vapors of a pH 1.5 HCl solution at 60 °C. SEM images of (j) ACR-, (k) ACR–BTA-, and (l, m) ACR–MSN–BTA–Ag-coated iron disks after 18 h of exposition to vapors of a pH 1.5 HCl solution at 60 °C. (n) EDX results collected on bright nanoparticles evidenced in panel (m).

To test the efficiency of the smart nanocarriers in acid conditions, the coated mock-up samples were exposed to vapors of a pH 1.5 HCl solution at 60 °C. No appreciable differences among the disks covered with the commercial product (ACR), the product loaded with additional free BTA (ACR–BTA), and the product loaded with the smart MSN–BTA–Ag nanocarrier system (ACR–MSN–BTA–Ag) are recorded up to 5 h (Figure 5a–c). Then, after 8 h of exposure to acid vapors, the ACR disk starts to look noticeably more corroded than the others, with the appearance of 300–400 μm large dark areas (Figure 5d). At the same exposure time, the ACR–BTA rebar sample starts to show the first corrosion spots of 120–150 μm diameter, while the rebar coated with the smart ACR–MSN–BTA–Ag coating shows the presence of very limited corrosive phenomena (Figure 5e,f).

Indeed, the ACR–MSN–BTA–Ag disk does not show significant signs of corrosion up to 18 h, when the first corroded areas appear. At the same time, corrosion phenomena proceed on the ACR and ACR–BTA disks and the damaged areas are significantly larger (see Figure 5g–i). By image analysis, the area of the corroded zones in the optical images reported in Figure 5g–i was quantified, representing 4.1, 3.1, and 0.7% of the total areas of the ACR, ACR–BTA, and ACR–MSN–BTA–Ag disks, respectively. SEM analysis confirms these results: the surfaces of the ACR and ACR–BTA samples are widely covered by corrosion products (identified as iron oxides by EDX), in large aggregates of 30–60 μm width in the ACR sample and in smaller aggregates of 10–30 μm width in the ACR–BTA sample (Figure 5j,k). At the same time, only isolated corrosion spots with width less than 5 μm are evidenced on the ACR–MSN–BTA–Ag iron rebar samples (Figure 5l). Moreover, the effectiveness of the chlorine capturing by silver ions in the ACR–MSN–BTA–Ag sample is clearly confirmed in the coatings by combined SEM and EDX analyses (Figure 5m,n). The presence of AgCl nanoparticles located close to the MSN, revealed as bright nanostructures with lateral size lower than 20 nm and whose composition is verified by EDX (Figure 5n), confirms the hypothesized chlorine capture mechanism. Once the BTA–Ag complex is dissolved and silver ions, released together with BTA molecules, get in contact with chloride ions, they precipitate in very small silver chloride nanoparticles. Therefore, results demonstrate the long-lasting efficiency of the protective coating containing the smart nanocarriers MSN–BTA–Ag, which inhibits the corrosion phenomena up to about 18 h in the tested conditions, while the presence of free corrosion inhibitor in the same polymer coating inhibits corrosion up to only 8 h. This enhanced protection is to be ascribed to both the sequestration of permeating chloride ions, which are delayed and do not reach the metal substrate, and to the modulated release of BTA from the nanocarriers, which act as a reservoir of the corrosion inhibitor, hosting and protecting it,^9^ and releasing it under stimuli. Indeed, the MSN–BTA–Ag particles embedded in the ACR–MSN–BTA–Ag coating release the needed BTA to effectively contrast the corrosion phenomena. In this way, the triggered behavior of the smart MSN–BTA–Ag nanocarriers constitutes an active response to the corrosion phenomena, thus ensuring longer protective efficiency in comparison to the polymer coating with the freely dispersed corrosion inhibitor.

**Figure 6:**
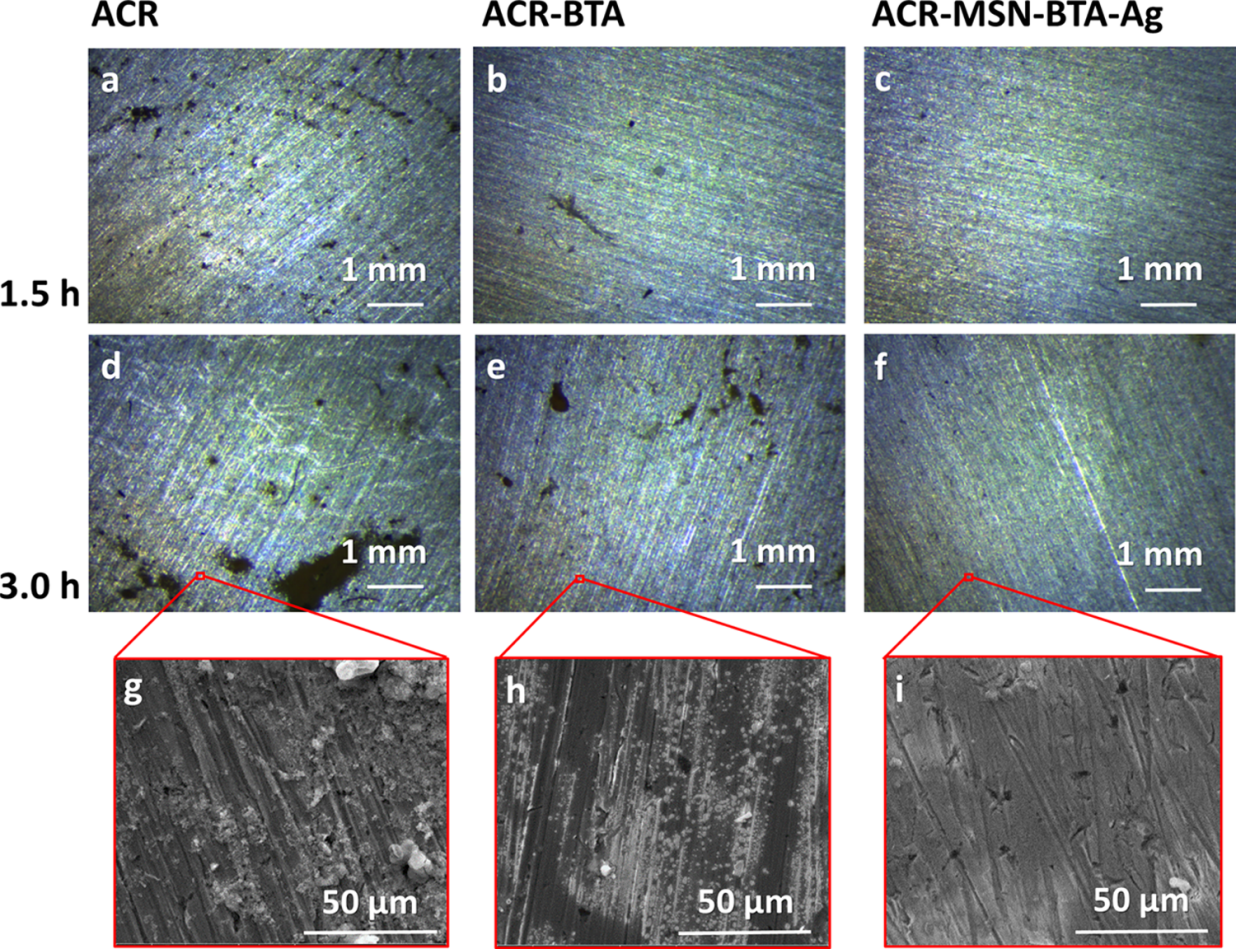
Optical microscopy images of (a,d) ACR-, (b,e) ACR–BTA-, and (c,f) ACR–MSN–BTA–Ag-coated disks after (a–c) 90 min and (d–f) 3 h of exposure to NaOH solution at pH 12.5 at 40 °C; SEM images of (g) ACR-, (h) ACR–BTA-, and (i) ACR–MSN–BTA–Ag-coated disks after 3 h of exposure.

The anticorrosive protection of the MSN–BTA–Ag nanocarriers was further tested in basic conditions. The ACR-, ACR–BTA-, and ACR–MSN–BTA–Ag-coated disks were treated with a pH 12.5 NaOH solution at 40 °C and monitored by optical microscopy. In this case, the ACR and the ACR–BTA disks show the first effects of corrosion after 90 min, with the appearance of 100–200 μm corroded areas, while the ACR–MSN–BTA–Ag-coated disk are still quite undamaged at the same treatment time (Figure 6a–c). After 3 h of exposure to NaOH, the first corrosion spots appear on the ACR–MSN–BTA–Ag-coated disk, while the corroded areas are significantly widened in the ACR and ACR–BTA-coated disks (Figure 6d–f). In this case, corroded areas in the images reported in Figure 6d–f are quantified as the 8.0%, 2.5%, and 0.1% of the total areas of the ACR-, ACR–BTA-, and ACR–MSN–BTA–Ag-coated disks, respectively. SEM analysis performed on the samples after 3 h of weathering in basic conditions confirms the large difference between the tested samples. Only small corrosion spots with width less than 5 μm are observed on the ACR–MSN–BTA–Ag-coated sample, while much larger corrosion areas are evidenced on the ACR- and ACR–BTA-coated disks (Figure 6g–i).

Also, in this case, the results demonstrate the major protective efficiency of the coating containing the smart nanocarriers. Indeed, although the alkaline testing conditions used are more aggressive than the acid ones, and the corrosion on all of these samples starts at a shorter time than in the acid tests, a significant difference is shown among the samples coated with the acrylic resin, the acrylic resin containing free BTA, and the samples coated with the resin containing the smart nanocarriers, which are able to release BTA on demand upon variable pH conditions.

The effect of the nanocarriers embedding BTA whose release is ruled by the silver capping layer was also compared to the effect of the BTA–Ag complex added to the commercial protective product in absence of the mesoporous silica nanoparticles (coated disk coded ACR–BTA–Ag). As shown in Figure S6, after the same treatments whose results are shown in Figures 5 and 6 for ACR, ACR–BTA, and ACR–MSN–BTA–Ag samples, the surfaces of ACR–BTA–Ag rebars appear very damaged. The extent of the corroded areas after acid and basic treatments are similar or lower than the corroded areas shown by the pristine commercial product (ACR) but much higher than the corroded areas shown by either the ACR–BTA and the ACR–MSN–BTA–Ag samples, confirming that the protective effect of MSN–BTA–Ag nanoparticles is to be ascribed to the combined effect of the smart nanocarriers acting as a long-lasting reservoir with the tailored triggered release of anticorrosion agents and not only to the combined effect of the BTA and silver ions.

**Figure 7:**
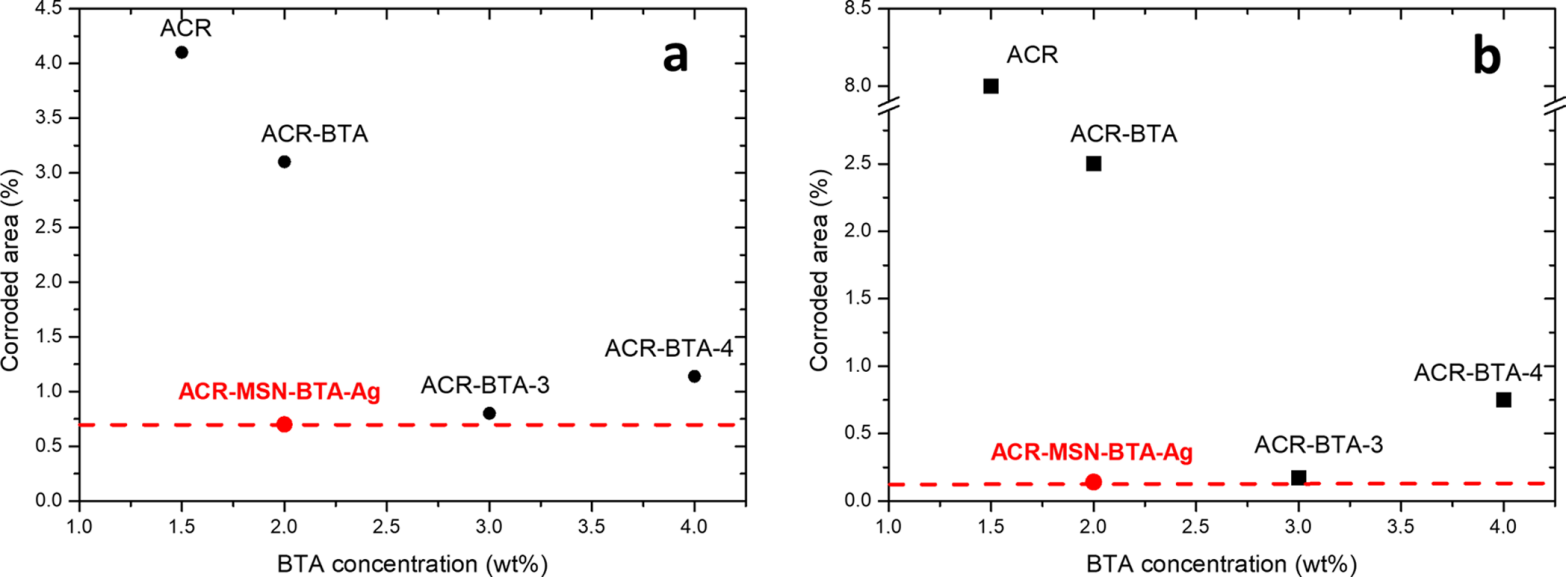
Corroded areas (%) of coated disks vs. total BTA concentration (free + loaded in MSN–BTA–Ag) for samples weathered in acid (a) and basic (b) conditions. Red dotted lines represent the extent of the corroded areas of the weathered ACR–MSN–BTA–Ag-coated samples.

Moreover, accelerated acid and basic corrosion tests using the same conditions already reported for ACR-, ACR–BTA-, and ACR–MSN–BTA–Ag-coated disks were performed on samples coated with the acrylic protective coating containing 3 and 4 wt % amounts of free BTA, namely, ACR–BTA-3 and ACR–BTA-4. The quantitative results are summarized in Figure 7. Characterization of these samples revealed that for ACR–BTA-3-coated disks, corroded areas covered 0.80 and 0.17% of the sample surface, after acid and basic exposure, respectively (Figure S7a,c). For ACR–BTA-4-coated disks, corroded areas increased to 1.14% in acid and 0.75% in alkaline conditions (Figure S7b,d). Therefore, the ACR–MSN–BTA–Ag coating provides a similar or higher protection to corrosion for the tested iron substrates in both acid and basic conditions than the acrylic coating containing free BTA, even when BTA is used at a concentration higher than 2 wt %. In particular, by increasing the amount of BTA to 3 wt %, the anticorrosion effectiveness of the coating to both acid and basic environments increases, and the extents of corroded areas in the tested conditions reach values close to those obtained with the ACR–MSN–BTA–Ag coating, but further increasing the amount of free BTA to 4 wt %, the coating efficiency slightly decreases again.

Thus, the MSN–BTA–Ag nanocarriers show an anticorrosion effect better than that shown by protective coatings containing much higher amounts of free BTA. To have further indication about the mechanisms through which MSN–BTA–Ag nanoparticles are able to protect the substrate, SEM analyses on unaged coatings ACR–BTA, ACR–MSN–BTA–Ag, ACR–BTA-3, and ACR–BTA-4 were performed to investigate the capability of BTA to form a continuous hydrophobic layer onto the metal substrate. Results shown in Figure S8 reveal that BTA molecules tend to agglomerate at a high concentration, as shown for the ACR–BTA-3 and ACR–BTA-4 coatings. These agglomerates prevent the formation of a continuous protective layer and represent defective points of the coating, whose presence explains the lower anticorrosion protection provided by ACR–BTA-4. On the contrary, BTA agglomerates are not evidenced in either ACR–BTA or ACR–MSN–BTA–Ag coatings. Therefore, embedding MSN–BTA–Ag nanoparticles in the polymer coating allowed to overcome the BTA agglomeration issue and at the same time obtain a higher protection efficiency.

Moreover, the evidence that larger amounts of free BTA are not useful to improve the protection efficiency of the substrate is supported also by theoretical considerations. The theoretical amount of BTA needed to totally cover the disk surface with a single BTA layer depends on the interaction of BTA molecules with the iron-based surface. This interaction has been largely reported in the literature, but the determination of the precise configuration of the complex is still uncertain.^53^ In particular, depending on the metal, BTA is able to interact with the metals atoms on the surface through different configurations.^54^ Reported BTA–Fe configurations suggest that the density of BTA molecules on the iron surface can be higher than in other BTA–metal complexes, such as copper or aluminum.^51,55^ Based on the hypothesized BTA–Fe structures,^41,51,56,57^ a BTA molecule could locate slightly sloped to the iron surface, due to the partial opening of the double bond between the nitrogen atoms, which allows their interaction with two consecutive Fe(II) ions on the substrate (see Figure S9). Considering the length of BTA molecule of about 0.6 nm and approximating its thickness to the atomic dimension, about 0.1 μg of BTA was needed to entirely cover a 1.2 cm diameter disk. This means that a 2 μm thick ACR–BTA coating, in which about 1 μg of BTA is embedded, already contains enough BTA to form a continuous protective multilayer adsorbed onto the iron surface.^48^

**Figure 8:**
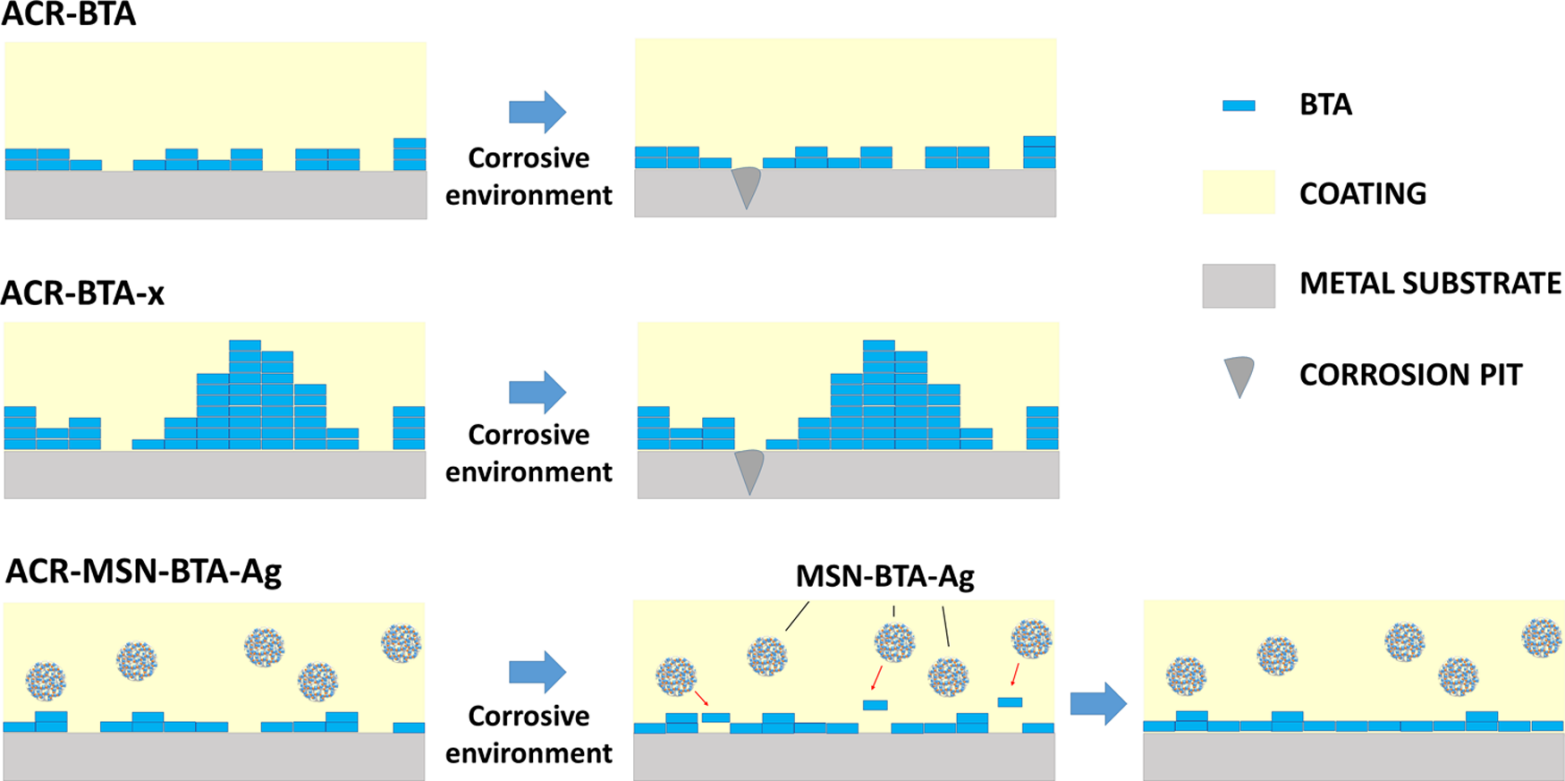
Schematic representation of the corrosion protection and failure mechanisms of the coatings ACR–BTA, ACR–BTA-x (i.e., ACR–BTA-3 or ACR–BTA-4), and ACR–MSN–BTA–Ag.

Based on these overall results and considerations, a possible schematic representation of the corrosion protection mechanism of ACR–MSN–BTA–Ag in comparison to a coating containing only free BTA is reported in Figure 8. The increase of BTA amount in the polymeric coatings does not ensure the formation of continuous barrier layers of anticorrosive agents on the metal surface, as most of BTA molecules aggregate, preventing the repair of the anticorrosive layer when corrosion starts. On the contrary, the higher protective efficiency of the ACR–MSN–BTA–Ag coating is well explained by the slow, smart release of BTA, which starts when the first aggressive species penetrate into the coatings and allow BTA diffusion through the coating, realizing an effective protective layer on the iron surface and minimizing the aggregation effect.

Thus, the MSN–BTA–Ag nanocarriers allow a tailored release of BTA, which can effectively prevent pit formation in metal structures. An excess of BTA in the coating matrix is not able to ensure a comparable protective effect, due to the BTA aggregation tendency at higher concentrations. On the contrary, the tailored release of the anticorrosive agent from the MSN–BTA–Ag nanocarriers prevents the formation of aggregates, guaranteeing a better formation of the protective layers with a higher and long-lasting anticorrosive efficiency.

## Conclusions

In this work, new smart corrosion inhibitor nanocarriers based on high-surface-area functional mesoporous silica nanoparticles, were developed. MSNs were obtained through a high-yield–high-throughput synthesis and loaded with the corrosion inhibitor BTA through an optimized procedure. Then, a new pH-dependent capping system based on a BTA–silver complex able to respond to both acid and alkaline triggers was designed and developed. In details, MSN–BTA–Ag nanocarriers were exploited to realize a multifunctional smart coating with a twofold anticorrosive mechanism in aggressive conditions involving Cl^–^: a passive mechanism, based on the triggered release of BTA, which creates a protective layer adsorbed on the metal surface, and an active mechanism, ascribed to the silver capability to sequestrate chloride ions and thus to delay the permeation of aggressive species toward the metal surface.

All results demonstrated the following:

(A) The BTA–Ag coordination complex is stable in neutral pH conditions and is able to tailor the BTA release in a wide pH range.
(B) Silver ions are effective in sequestrating the chloride ions, thus contributing to increase in the anticorrosive efficiency of the coatings.
(C) The exploitation of the concept of nanocarrier is an effective strategy to improve the anticorrosive protection of coatings, due to the on-demand release of BTA, which allows avoiding the formation of large BTA aggregates in the coatings and realizing a better covering of the metal substrates.

## Supporting Information Available

Additional experimental details, materials and methods, additional results, figures, and tables: (S1) BTA–Ag morphological and FTIR analysis and solubility tests; (S2) Comparison of color between MSN–BTA–Cu and MSN–BTA–Ag; (S3) EDX analysis of MSN–BTA–Ag and MSN; (S4) untreated and uncoated rebar iron disk; (S5) acid and alkaline environment exposure of ACR–BTA–Ag-coated disks; (S6) acid and alkaline environment exposure of ACR–BTA-3- and ACR–BTA-4-coated disks; (S7) evaluation of BTA aggregation tendency; and (S8) interaction between BTA and an iron substrate (PDF)
