# Supplementary material for: Innovative Silver-Based Capping System for Mesoporous Silica Nanocarriers Able to Exploit a Twofold Anticorrosive Mechanism in Composite Polymer Coatings: Tailoring Benzotriazole Release and Capturing Chloride Ions
Source: ACS Appl Mater Interfaces. 2021 Oct 5;13(40):48141–52. doi: 10.1021/acsami.1c15231 (PMC9282642; doi:10.1021/acsami.1c15231)
Supplement: Supplementary file 1 — am1c15231_si_001.pdf [file am1c15231_si_001.pdf]

Innovative silver-based capping system for mesoporous silica nanocarriers able to exploit a two-fold anticorrosive mechanism in composite polymer coatings: tailoring benzotriazole release and capturing chloride ions

Federico Olivieri[a], Rachele Castaldo[a], Mariacristina Cocca[a], Gennaro Gentile*[a], Marino Lavorgna[b]*

[a] Institute for Polymers, Composites and Biomaterials, National Research Council of Italy Via Campi Flegrei, 34, 80078 Pozzuoli, Italy.

[b] Institute for Polymers, Composites and Biomaterials, National Research Council of Italy P.le E. Fermi 1, 80055 Portici, Italy.

Supporting Information

S1. BTA-Ag morphological and FTIR analysis and solubility tests

S2. Comparison of color between MSN-BTA-Cu and MSN-BTA-Ag

S3. EDX analysis on MSN-BTA-Ag and MSN

S4. Untreated and uncoated rebar iron disk

S5. Acid and alkaline environment exposure of ACR-BTA-Ag coated disks

S6. Acid and alkaline environment exposure of ACR-BTA-3 and ACR-BTA-4 coated disks

S7. Evaluation of BTA aggregation tendency

S8. Interaction between BTA and an iron substrate

S1. BTA-Ag morphological and FTIR analysis and solubility tests

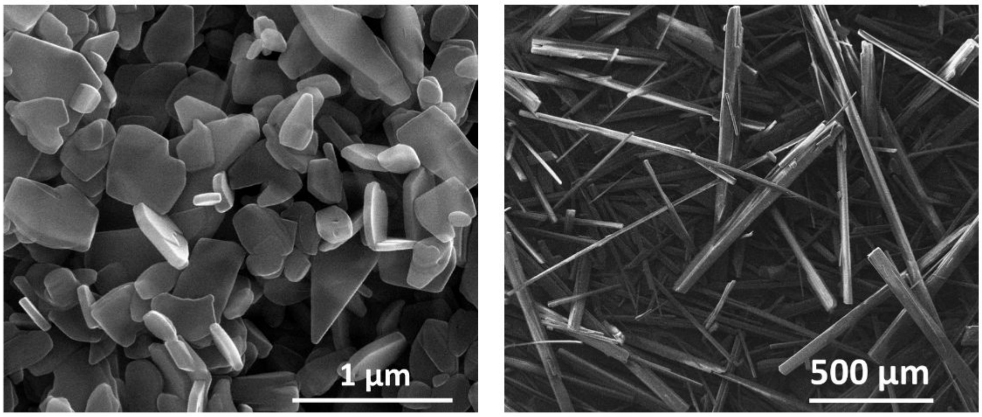

Figure S1. SEM micrographs of the BTA-Ag complex (left) and the pristine BTA (right).

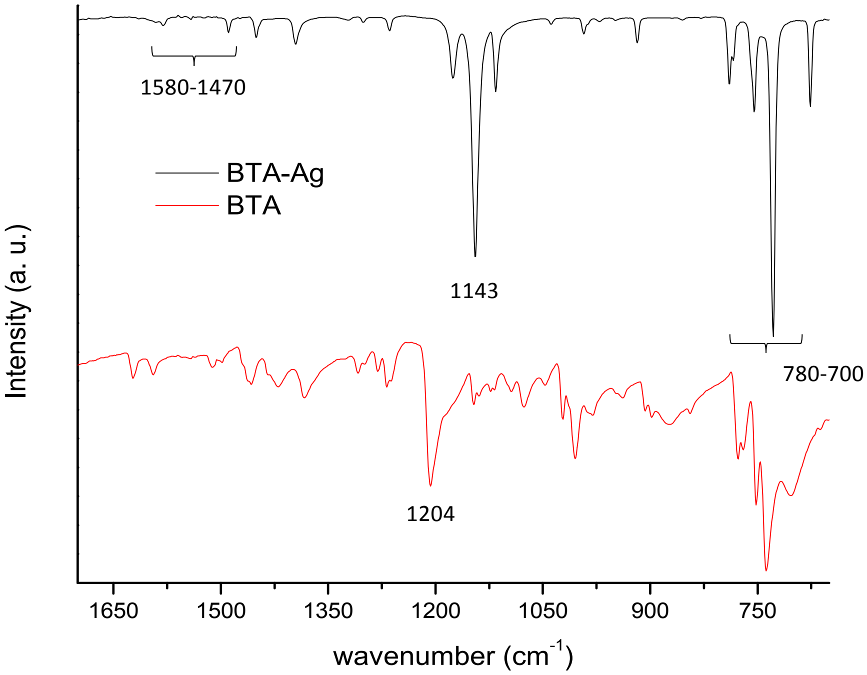

Figure S2. ATR-FTIR spectra of the BTA-Ag complex and the pristine BTA in the range between 1700 and 650 cm-1.

Solubility tests revealed a low solubility at pH 5, 6 and 9 and almost insolubility at pH 7 and 8. Results are shown in Figure S4. At pH 5, dissolved BTA was up than 70 mg/L, while at pH 6 and 9 the solubility decreased to about 35 mg/L. At pH 7 and 8 BTA-Ag solubility was less than 15 mg/L, confirming the very low BTA-Ag solubility in this range.

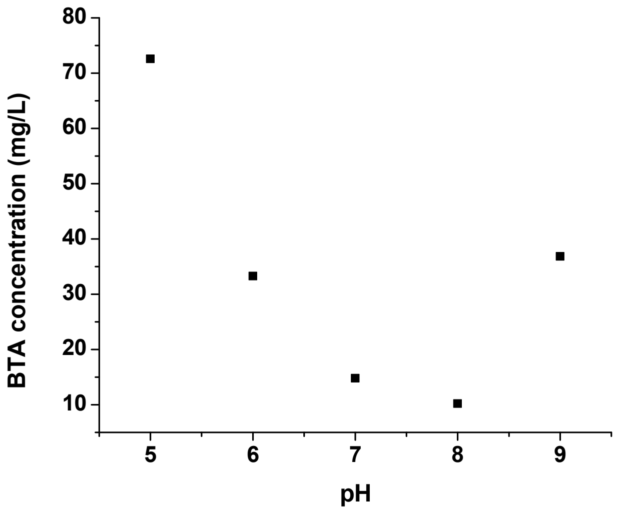

Figure S3. Solubility of BTA-Ag at pH 5, 6, 7, 8 and 9 (total content of BTA 220 mg/L).

S2. Comparison of color between MSN-BTA-Cu and MSN-BTA-Ag

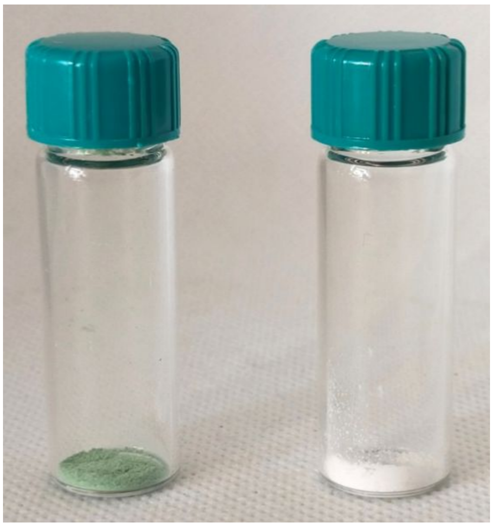

Figure S4. Green-blue colored MSN-BTA-Cu (left) and white MSN-BTA-Ag (right).

S3. EDX analysis on MSN-BTA-Ag and MSN

Table S1. Atomic composition of MSN-BTA-Ag and pristine MSN

Sample	Elemental content (atomic %)			
	C	O	Si	Ag
MSN-BTA-Ag	22.00 ± 2.22	53.93 ± 0.79	21.14 ± 1.78	2.93 ± 0.54
MSN	-	71.42 ± 2.67	28.18 ± 2.12	-

S4. Untreated and uncoated rebar iron disk

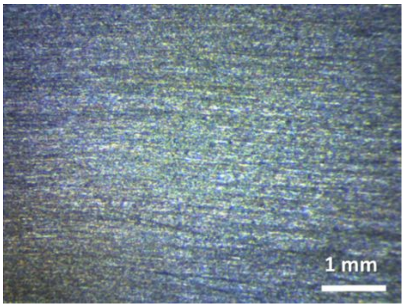

Figure S5. Optical images of an untreated and uncoated rebar iron disk.

S5. Acid and alkaline environment exposure of ACR-BTA-Ag coated disks

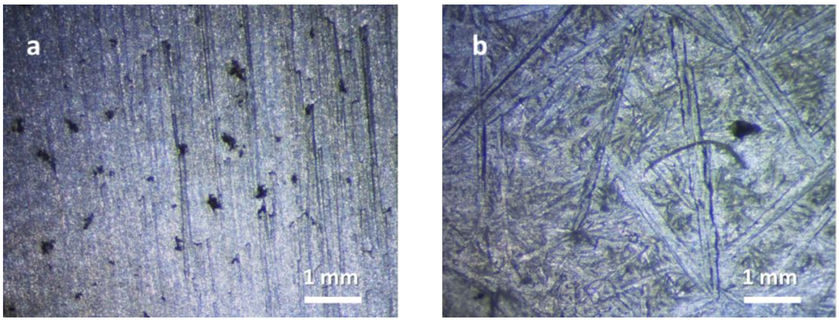

Figure S6. Optical images of ACR-BTA-Ag coated disks after 3 hours of HCl vapor exposition (pH 1.5) at 60˚C (a) and 18 h of direct contact with NaOH water solution (pH 12.5) at 40 ˚C (b).

S6. Acid and alkaline environment exposure of ACR-BTA-3 and ACR-BTA-4 coated disks

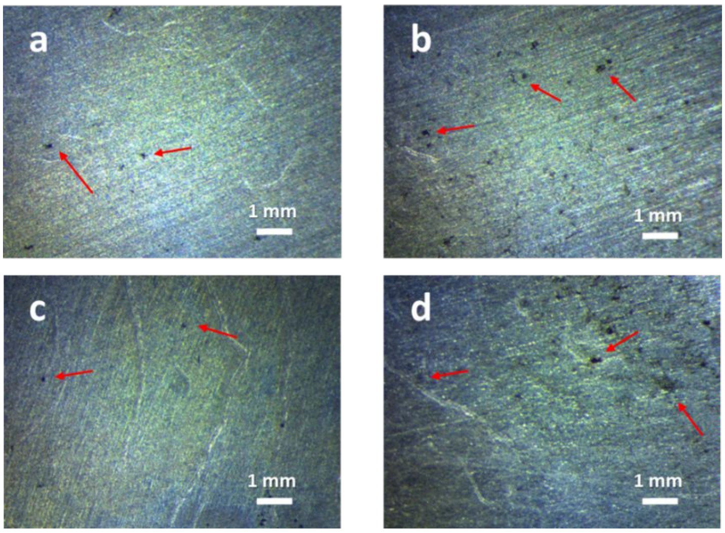

Figure S7. Optical images of ACR-BTA-3 (a,c) and ACR-BTA-4 (b,d) coated disks after 3 hours of HCl vapor exposition (ph 1.5) at 60°C (a,b) and 18 h of direct contact with NaOH water solution (ph 12.5) (c,d). Red arrows indicate corrosion pits.

S7. Evaluation of BTA aggregation tendency

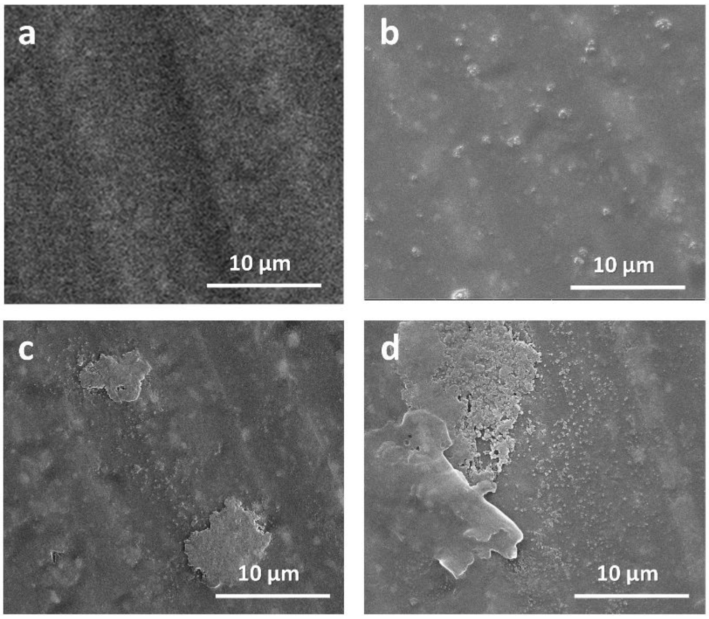

Figure S8. SEM micrographs of ACR-BTA (a), ACR-MSN-BTA-Ag (b), ACR-BTA-3 (c) and ACR-BTA-4 (d) coatings.

S8. Interaction between BTA and an iron substrate

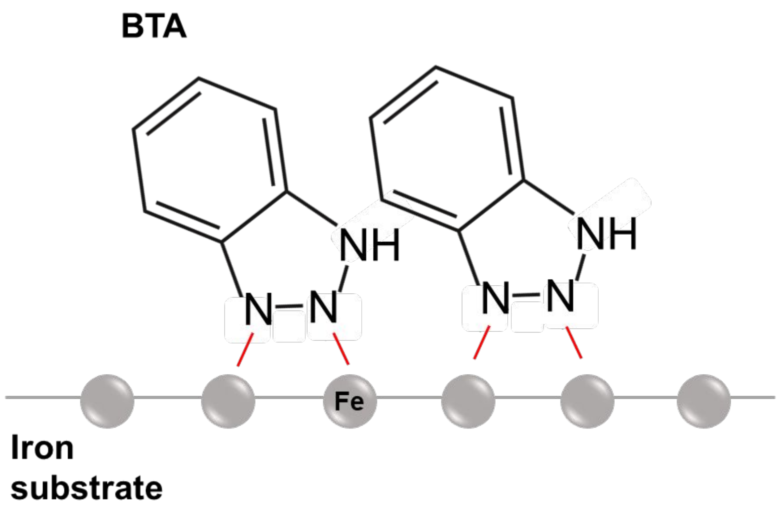

Figure S9. BTA-Fe complex structure on iron substrate.
